# Changes in Membrane Protein Clustering in Peripheral Lymphocytes in an Animal Model of Depression Parallel Those Observed in Naïve Depression Patients: Implications for the Development of Novel Biomarkers of Depression

**DOI:** 10.3389/fphar.2018.01149

**Published:** 2018-10-15

**Authors:** Raquel Romay-Tallon, Erin Kulhawy, Kyle J. Brymer, Josh Allen, Tania Rivera-Baltanas, Jose M. Olivares, Lisa E. Kalynchuk, Hector J. Caruncho

**Affiliations:** ^1^Division of Medical Sciences, University of Victoria, Victoria, BC, Canada; ^2^Innovate-Calgary, University of Calgary, Calgary, AB, Canada; ^3^Department of Psychology, University of Saskatchewan, Saskatoon, SK, Canada; ^4^Division of Psychiatry, Hospital Alvaro Cunqueiro, CHUVI, Vigo, Spain

**Keywords:** depression, corticosterone, lipid rafts, biomarkers, lymphocytes

## Abstract

Naïve depression patients show alterations in serotonin transporter (SERT) and serotonin 2A (5HT2A) receptor clustering in peripheral lymphocytes, and these alterations have been proposed as a biomarker of therapeutic efficacy in major depression. Repeated corticosterone (CORT) induces a consistent depression-like phenotype and has been widely used as an animal model to study neurobiological alterations underlying the depressive symptoms. In this experiment, we used the CORT paradigm to evaluate whether depression-like behavior is associated with similar changes in the pattern of SERT and 5HT2A membrane protein clustering as those observed in depression patients. We also analyzed the clustering of other proteins expressed in lipid rafts in lymphocytes. Rats received daily CORT or vehicle injections for 21 consecutive days. Afterward they underwent the forced swim test to evaluate depression-like behavior, and isolated lymphocytes were analyzed by immunocytochemistry coupled to image-analysis to study clustering parameters of the SERT, 5HT2A receptor, dopamine transporter (DAT), Beta2 adrenergic receptor (β2AR), NMDA 2B receptor (NR2B), Pannexin 1 (Pnx1), and prion cellular protein (PrPc). Our results showed that CORT increases the size of protein clusters for all proteins with the exception of β 2AR, which is decreased. CORT also increased the number of clusters for Pnx1 and PrPc only. Overall, these results indicate that alterations in SERT and 5HT2A protein clustering in naïve depression patients are paralleled by changes seen in an animal model of depression. The CORT paradigm may be a useful screen for examining additional proteins in lymphocytes as a preliminary step prior to their analysis as biomarkers of depression in human blood samples.

## Introduction

Chronic stress and associated inflammatory events have been repeatedly shown to play a major role in the pathophysiology of depression (recently reviewed by Leonard, 2018). In fact several meta-analyses have revealed a consistent increased expression of pro-inflammatory cytokines – particularly IL-6, TNF-α, and CRP- in clinical depression (see Felger and Lotrich, 2013; Strawbridge et al., 2015; Haapakoski et al., 2016), and alterations in peripheral proteins related to inflammatory events have also been evaluated as possible biomarkers of depression (recently reviewed by Gururajan et al., 2016; Gadad et al., 2018). Also of importance in the context of the psychoneuroimmunology of stress/depression is the finding that high levels of stress cause a reduction in T and B lymphocyte proliferation, a reduction in immunoglobulin production, and an increase in neutrophils and macrophage activation and spreading (Dhabhar, 2008; Divyashree et al., 2016). Finally, we have recently reported that changes in the clustering of serotonin transporter (SERT) and serotonin 2A receptor (5HT2A) membrane proteins in lymphocytes in naïve depression patients give indications about the degree to which these patients will respond to antidepressant medication. These latter results have led us to propose that the pattern of clustering of these proteins could be a novel biomarker of therapeutic efficacy in major depression (Rivera-Baltanas et al., 2012, 2014, 2015).

Unsurprisingly, rodents subjected to different forms of chronic stress have consistently shown depression-like behavior and have been widely used to analyze the neurobiological events underlying the onset of a depressive phenotype (reviewed by Sterner and Kalynchuk, 2010). One well-characterized model makes use of repeated injections of corticosterone (CORT), which results in a robust and reliable increase in depression-like behavior as ascertained by different behavioral paradigms (Kalynchuk et al., 2004; Gregus et al., 2005; Marks et al., 2009), and a concomitant decrease in both adult hippocampal neurogenesis and expression of the extracellular matrix protein reelin in the proliferative subgranular zone of the dentate gyrus (Lussier et al., 2009, 2011, 2013). There appears to be a strong link between reelin and neurogenesis in the context of depression-like behavior. In addition, reelin is also involved in dendritogenesis, spinogenesis, and regulation of LTP, which are other forms of plasticity altered in depression, thereby indicating the interest in studying both central in peripheral actions of reelin within the context of depression, and fostering the interest in the study of the possible antidepressant-like characteristics of reelin peptides (Caruncho et al., 2016).

Following our studies in patient with depression, we now hypothesize that CORT will induce alterations in SERT and 5HT2A clustering along the cell membrane of peripheral lymphocytes that parallel those observed in human patients (Rivera-Baltanas et al., 2012, 2014). If so, and as these two proteins tend to cluster in specific microdomains such as lipid rafts (Magnani et al., 2004; Allen et al., 2007) and as interaction of antidepressants with lipid rafts is thought to be an important functional link for antidepressant efficacy (Donati and Rasenick, 2005; Zhang and Rasenick, 2010; Czysz and Rasenick, 2013; Czysz et al., 2015; Donati et al., 2015; Erb et al., 2016), we may further hypothesize that clustering of other membrane proteins that tend to accumulate in lipid rafts may be significantly changed in lymphocytes upon repeated CORT treatment, and that some parameters of alterations in membrane protein clustering may correlate with depression-like behavior as evaluated by the forced swim test (FST). As such, we also included in this experiment the analysis of membrane protein clustering for the dopamine transporter (DAT), the beta 2 adrenergic receptor (β2AR), the NMDA receptor 2B subunit (NR2B), pannexin 1 (Pnx1), and the prion cellular protein (PrPc). This experimental design will bring about our focus on the most translational component of our research, as will focus first in demonstrating if one can find in the CORT model of depression changes in the clustering pattern of SERT and 5HT2A that are similar to those found in naïve depression patients, and if so use this approach to analyze the patterns of clustering of other proteins that can thereby be screened for further analysis in depression patients (using a bedside to bench to bedside approach).

## Materials and Methods

### Animals

We used a total of 53 adult male Long Evans rats in this experiment (purchased from Charles River Laboratories, Montreal, Quebec). Rats weighed 200–250 g at the time of arrival (rats were reared under conventional conditions by the breeder) and were single-housed with food and water provided *ad libitum*. The colony was kept under controlled conditions of light and temperature (12 h:12 h light-dark cycle, 21 ± 1°C). All the experimental procedures were conducted under protocol #20140038, approved by the Animal Research Ethics Board of the University of Saskatchewan.

About half of the animals were injected with vehicle and the other half with CORT as explained below, and thereafter they were evaluated for depression-like behavior in the FST. A set of 20 rats (10 vehicle, 10 CORT) was used to examine SERT clustering to compare with our previous analyses of SERT in patients with depression (Rivera-Baltanas et al., 2012, 2015); a set of 17 rats (9 vehicle, 8 CORT) was used to examine 5HT2A and β2AR clustering, to compare with previous studies on 5HT2A in depression patients (Rivera-Baltanas et al., 2014), and to analyze an additional G-protein coupled receptor; and a finals set of 16 rats (8 vehicle, 8 CORT) was used to examine novel membrane proteins (DAT, NR2B, Pnx 1, and PrPc) that could then be screened for further studies of membrane protein clustering in depression patients.

### Corticosterone Treatment

Rats were handled briefly once per day for 7 days prior to the CORT or vehicle injections. Following this acclimatization period, rats were weight-matched into two groups that received either 21 days of CORT injections (CORT group) or 21 days of vehicle injections (vehicle group). All injections were administered subcutaneously once per day (between 9:00 and 2:00 pm). CORT (Steraloids) was suspended in 0.9% (w/v) physiological saline with 2% (v/v) Tween-80 and given at a dose of 40 mg/kg in a volume of 1 ml/kg. Previous work in our laboratory and others has demonstrated that the dose of 40 mg/kg of CORT reliably increases depression-like behavior in rats (Kalynchuk et al., 2004; Marks et al., 2009; Fenton et al., 2015; Kott et al., 2016; Workman et al., 2016).

### Forced Swim Test

The FST was conducted the day after the final CORT or vehicle injection. We used a modified version of the traditional Porsolt test (Porsolt et al., 1977). This version of the test is conducted over 1 day, which is as effective as 2-day version for assessing depression-like behavior in rats previously subjected to a period of chronic stress (Marks et al., 2009).

The FST was carried out in a different room from the one used for the CORT injections. Rats were placed into a rectangular Plexiglas swim tank (25 cm long × 25 cm wide × 60 cm high) filled with water to a depth of 30 cm. The temperature of the water was kept at 27°C (±2°C). Rats remained in the tank for 10 min and the session was videotaped for offline analyses. We scored both active and inactive components of behavior: swimming, struggling, and immobile (Marks et al., 2009).

### Extraction of Lymphocytes and Immunocytochemistry

Rats were deeply anesthetized with isoflurane. Thereafter, six ml of blood were collected by heart puncture with ACD anticoagulant (85 mM trisodium citrate, 65 mM citric acid, 111 mM anhydrous glucose) at a ratio 1:7 (v/v). Blood was diluted 1:1 with phosphate buffer saline (PBS) and centrifuged in a Percoll gradient for 40 min at 800 × *g*. The lymphocyte band was then collected and cells were re-suspended in PBS solution and centrifuged at 1000 × *g* for 10 min. This step was repeated twice. Lymphocyte fixation involved incubation with 1% paraformaldehyde in 0.1 M phosphate buffer (PB) for 5 min (Rivera-Baltanas et al., 2010; Romay-Tallon et al., 2017).

Immunolabeling of specific membrane proteins was used to enable to assessment of membrane protein clustering. Prior to immunolabeling, we performed a preincubation step to avoid unspecific staining by incubating with a blocking solution [3% rat IgG (Sigma) and 1% bovine serum albumin (BSA)] in PBS for 10 min at room temperature. This was followed by overnight incubation at 4°C with a series of rabbit-polyclonal primary antibodies diluted in blocking solution: anti-SERT (1:250, Cat# AB10514P, Millipore), anti-5HT2A (1:150, Cat# RA24288, Neuromics); anti-β2AR (1:150, Cat# SAB1306036, Sigma); anti-PrPc (1:200, Cat# ab52604, Abcam); anti-NR2B (1:200, Cat# SAB4300711, Sigma); anti-DAT (1:250, Cat#D6944, Sigma); anti-Pannexin1 (PNX-1, 1:250, Cat#ab124131, Abcam).

The binding of the primary antibodies to the antigen was then revealed by incubation with a secondary antibody, goat anti rabbit Alexa Fluor 568 (1:200, Molecular Probes) diluted in 1% BSA in PBS for 1 h at room temperature. Several washes in PBS were performed after finishing the incubation with the secondary antibody, and thereafter samples were collected onto microscope slides and cover-slipped with Citifluor (Electron Microscope Science). Samples were stored at -20°C until analysis.

Omission of primary antibodies resulted in lack of immunostaining in all cases.

### Imaging and Statistical Analysis

A total of 50 lymphocytes per animal were analyzed with a Nikon E800 microscope using computer software (MicroBrightfield) with a MicroFire digital camera (Optronics). The number and size of protein clusters were analyzed with ImageJ 1.48 software (NIH), using previously published methods (Romay-Tallon et al., 2017).

All data were analyzed with SPSS (Statistical Package for the Social Science, v16.0, Chicago). We used independent sample *t*-tests to evaluate differences between the vehicle and CORT groups in behavior and protein clustering. The data are expressed as the mean value ± SEM. Differences were considered statistically significant at *p* < 0.05.

## Results

### CORT-Treated Rats Show Depression-Like Behavior in the FST

**FIGURE 1:**
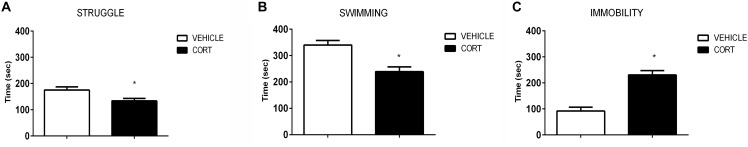
Results of the forced swim test demonstrate that repeated CORT induces depressive-like behavior, as evidenced by analysis of the struggle **(A)**, swimming **(B)**, and immobility time **(C)**. Significant differences, *p* value < 0.05, are indicated by an asterisk (^∗^).

**Figure 1** shows the effect of CORT on FST behavior. As expected, rats injected with CORT spent less time active than their vehicle counterparts. *T*-test analyses revealed a significant increase in the time the CORT rats spent immobile relative to the vehicle rats [*t*(51): -6.140; *p* = 0.0000001], and a decrease in the amount of time spent swimming [*t*(51): 4.108; *p* = 0.0001], and struggling [*t*(51): 2.591; *p* = 0.012].

### Serotonin Transporter

**FIGURE 2:**
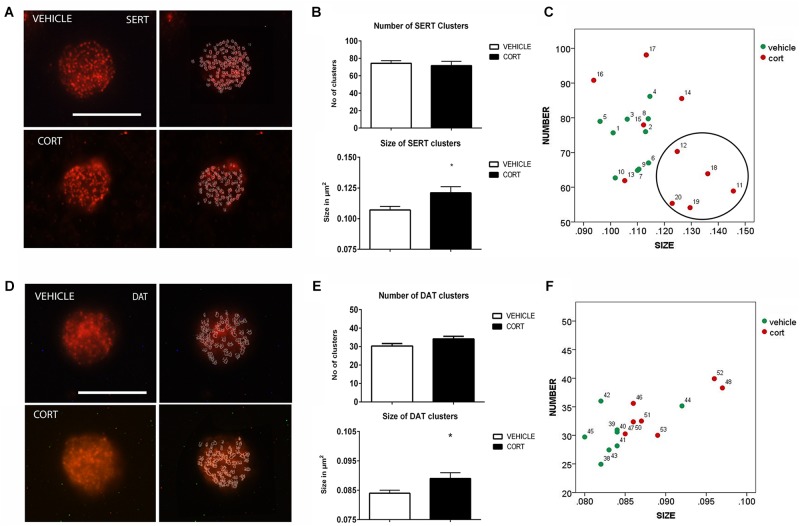
Analysis of SERT and DAT MPC in lymphocytes. SERT immunolabeling is observed as patches of immunostaining in the plasma membrane of lymphocytes that are amenable to quantification by image analysis. Panel **(A)** shows SERT immunostaining in a representative lymphocyte from vehicle or CORT treated rats, and how these clusters are evaluated by using image-J. Calibration bar: 10 μm. Panel **(B)** shows that repeated CORT increases SERT clusters size but does not change the number of clusters per lymphocyte. Panel **(C)** left portraits the representation of the average number and size of SERT clusters in individual animals (identified by numbers). Note that a subset of CORT treated animals is identified by having a low number and high size of SERT clusters (circle). DAT immunolabeling is observed as patches of immunostaining in the plasma membrane of lymphocytes that are amenable to quantification by image analysis (Panel **D** Calibration bar: 10 μm). Panel **(E)** shows that repeated CORT increases DAT clusters size but does not change the number of clusters per lymphocyte. Panel **(F)** portraits a scatter plot of how CORT alters DAT MPC parameters. Significant differences, *p* value < 0.05, are indicated by an asterisk (^∗^).

**Figure 2A** shows representative images of lymphocytes from vehicle and CORT rats, stained for SERT. The imaging system creates an outline representation of clusters (**Figure 2A**, right column) allowing for the quantification of the number and size of SERT clusters. This quantification is shown in **Figure 2B**. *T*-test analyses revealed no differences between the groups in the number of clusters [*t*(18): 0.343, *p* = 0.736]. However, there was an increase of 13% in clusters size in CORT rats relative to vehicle-treated rats [*t*(18): -2.458, *p* = 0.030]. **Figure 2C** further illustrates the distribution of SERT clusters based on their number and size, with numbers in the graph indicating individual animals. This analysis reveals that a number of CORT rats showed a low number and high size of SERT clusters in lymphocytes (indicated with a circle in the left panel of **Figure 2C**), interestingly these rats showed a higher level of immobility in the FST which parallels observations in SERT clustering in naïve depression patients (see section “Discussion” and Rivera-Baltanas et al., 2012).

### Dopamine Transporter

As we demonstrated that changes in SERT clustering in lymphocytes from CORT treated animals parallel those observed in naïve depression patients, we proceeded to analyze other neurotransmitter transporter that is also related to depression, but whose clustering has not been studied yet in depression patients. **Figure 2D** shows the effect of CORT on DAT-positive protein clusters on the cell membrane of lymphocytes. We found a significant (7%) increase in the size of DAT clusters in the CORT rats relative to the vehicle rats [*t*(13): -2.505, *p*: 0.026], but no significant differences in the number of DAT clusters [*t*(13): -1.908, *p*: 0.079]. This is shown in **Figure 2E**. **Figure 2F** shows the relationship between clusters numbers and size.

### Serotonin Receptor 2A

**FIGURE 3:**
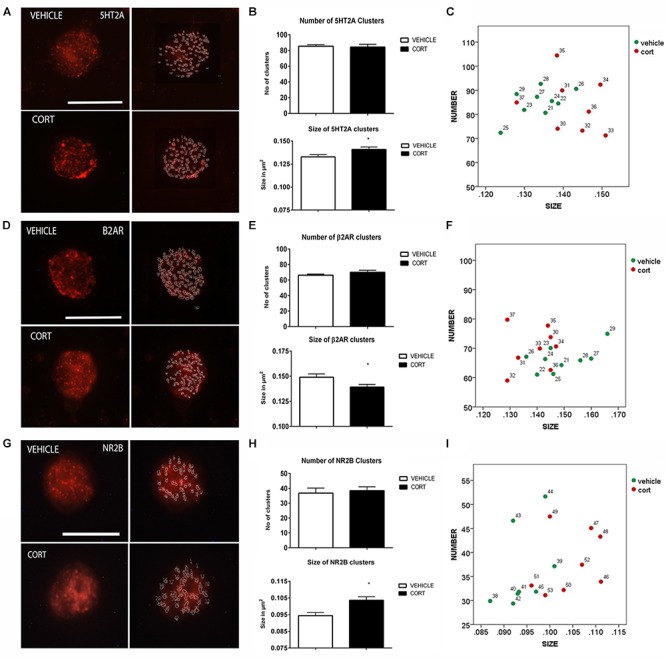
Analysis of 5HT2A, β2AR, and NR2B receptors MPC in lymphocytes. 5HT2A immunolabeling is observed as patches of immunostaining in the plasma membrane of lymphocytes that are amenable to quantification by image analysis (Panel **A** Calibration bar: 10 μm). Panel **(B)** shows that repeated CORT increases 5HT2A clusters size but does not change the number of clusters per lymphocyte, while Panel **(C)** left portraits the alterations induced by CORT in number Vs size of 5HT2A clusters. Panel **(D)** illustrates the identification of β2AR clusters in peripheral lymphocytes (Calibration bar: 10 μm). Panel **(E)** shows the effect of repeated CORT in decreasing the size of β2AR clusters without altering their number. Panel **(F)** portraits the representation of the average number and size of 5HT2A clusters in individual animals (identified by numbers). Note that while for most protein clusters CORT induces an increase in clusters size in the case of β2AR the size of the clusters is decreased. Panel **(G)** presents representative micrographs of NR2B labeling in lymphocytes from vehicle or CORT-treated animals (Calibration bar: 10 μm). Panel **(H)** shows the effect of repeated CORT in increasing the size of NR2B clusters without altering their number. Panel **(I)** portraits the representation of the average number and size of NR2B clusters in individual animals. Significant differences, *p* value < 0.05, are indicated by an asterisk (^∗^).

**Figure 3A** illustrates the immunolabeling of 5HT2A clusters in representative lymphocytes from the vehicle and CORT rats (left panels), as well as the cluster outlines revealed through the imaging system (right panels). Quantitative analysis showed that 5HT2A cluster size is increased 7% in the CORT rats [*t*(15): -2.568, *p* = 0.021] but there were no groups differences in the number of clusters [*t*(15): 0.218, *p*: 0.830] (**Figure 3B**). **Figure 3C** shows the distribution of 5HT2A clusters according to number and size in both groups. Similar changes have been previously observed in naïve depression patients (Rivera-Baltanas et al., 2014).

### Beta 2 Adrenergic Receptor

As changes in 5HT2A clustering in lymphocytes from CORT treated animals parallel those observed in naïve depression patients (see above), we proceeded to analyze other neurotransmitter receptors such as β2AR and NR2B, that are also related to depression, but whose clustering has not been studied yet in depression patients. **Figure 3D** shows the effect of CORT on β2AR protein clusters. Although there were no significant group differences in the number of clusters [*t*(15): -1.294, *p* = 0.215], there was a significant decrease (7%) in the size of the β2AR clusters in the CORT rats relative to the vehicle rats [*t*(15): 2.300, *p* = 0.036] (**Figure 3E**). **Figure 3F** shows the distribution of the clusters according with their number and size.

### NMDA Receptor 2B Subunit

**Figure 3G** shows the effect of CORT on NR2B-immunopositive protein clusters. There were no group differences in the number of NR2B clusters [*t*(14): -0.463, *p*: 0.650] but there was a significant increase in cluster size (i.e., 11%) in the CORT rats relative to the vehicle rats [*t*(14): -3.936, *p* = 0.001] (**Figure 3H**). **Figure 3I** represents the distribution of NR2B clusters based on number and size.

### Pannexin 1

**FIGURE 4:**
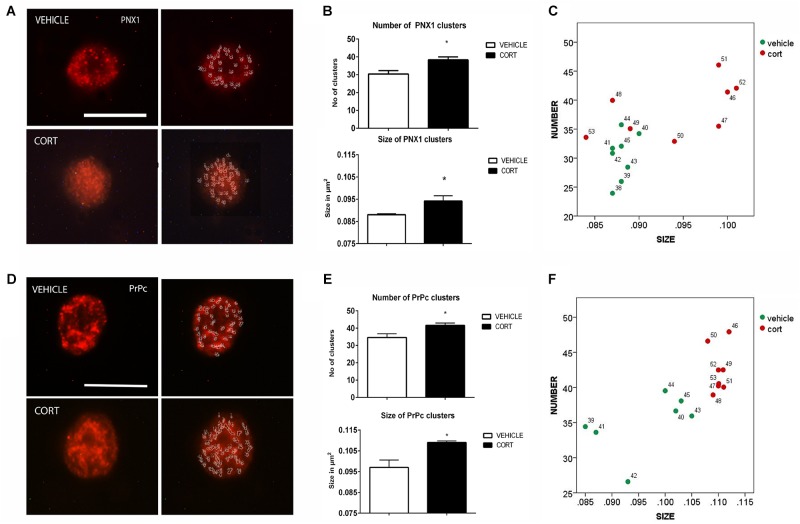
Analysis of Pnx1 and PrPc MPC in lymphocytes. Pnx1 immunolabeling is observed as patches of immunostaining in the plasma membrane of lymphocytes that are amenable to quantification by image analysis (Panel **A** Calibration bar: 10 μm). Panel **(B)** shows that repeated CORT increases both Pnx1 clusters number and size. Panel **(C)** illustrates changes in individual animals in relation to number of clusters Vs size. PrPc labeling micrographs are shown in Panel **(D)** (Calibration bar: 10 μm). Panel **(E)** illustrates the effect of repeated CORT in increasing both the number and size of PrPc clusters. Panel **(F)** portraits the representation of the average number and size of PrPc clusters in individual animals (identified by numbers). Significant differences, *p* value < 0.05, are indicated by an asterisk (^∗^).

On top of the analysis of the patterns of clustering of some neurotransmitter transporters and receptors we also proceeded to the analysis of other proteins that have been shown related to depression such as Pnx1 and PrPc, in order to ascertain the interest of further studying the patterns of clustering of these proteins in naïve depression patients. **Figure 4A** illustrates the effect of CORT on Pnx 1 immunopositive clusters along the cell membrane of lymphocytes. Quantification of Pnx 1 revealed a 26% increase in cluster number [*t*(14): -3.624, *p*: 0.003], and a 7% increase in cluster size [*t*(14): -2.592, *p*: 0.021] in the CORT rats relative to the vehicle rats (**Figure 4B**). The distribution of clusters based on the number and size is represented in **Figure 4C**.

### Prion Cellular Protein

**Figure 4D** shows the effect of CORT on PrPc protein clusters. Quantitative analyses of these clusters revealed an increase of 21 and 15% in the number and size of clusters in the CORT rats, respectively [Number: *t*(13): -3.852, *p*: 0.002; size: *t*(13): -4.756, *p* = 0.0003], see **Figure 4E**. **Figure 4F** shows the distribution of PrPc positive clusters.

## Discussion

To our knowledge, this is the first time that a panel of membrane protein clusters has been analyzed in peripheral mononuclear blood cells and analyzed in relation to depression-like behavior in an animal model for depression. Ultimately, we conducted this analysis with the idea that positive results could suggest the use of animal models such as the CORT paradigm to screen biomarkers of depression to be further studied in samples from depression patients, which are not always easy to obtain. The main findings of this experiment indicate in the first place that repeated CORT induces alterations in the pattern of SERT and 5HT2A clustering along the cell membrane of peripheral lymphocytes that parallel those observed in naïve depression patients (Rivera-Baltanas et al., 2012, 2014), and second it establishes how other membrane proteins related to depression changes their pattern of clustering in the CORT model thereby indicating that this model can be used as a screening tool to select specific proteins whose pattern of clustering would be of interest to further study in samples from depression patients.

The behavioral results obtained here are consistent with previous literature showing that repeated CORT administration reliably increases depression-like behavior (reviewed in Sterner and Kalynchuk, 2010). We found that CORT-treated rats showed increased immobility and decreased struggling and swimming in the FST, which is a pattern of behavior that generally indicates a depressive phenotype (Cryan et al., 2005). This result was expected based on previous findings showing that the 40 mg/kg dose increases immobility behavior in both male and female rats (Kalynchuk et al., 2004) and that the effects of CORT on immobility can be reversed by administration of imipramine – a tricyclic antidepressant (Fenton et al., 2015). Importantly, the depressogenic effects of CORT are not restricted to the FST. Rodents subjected to repeated CORT also show impaired cognition, decreased interest in sexual behavior, decreased sucrose preference, decreased home cage activity, and increased immobility in a tail suspension test (Gorzalka and Hanson, 1998; Dwivedi et al., 2015; Sturm et al., 2015; Huston et al., 2016; Lui et al., 2017; Brymer et al., 2018), without altering nonspecific motor behavior (Marks et al., 2009). However, one should point out that the FST is more a tool to screen antidepressant-like activity that not ascertain depressive-like behavior (Nestler and Hyman, 2010; Molendijk and de Kloet, 2015; de Kloet and Molendijk, 2016). This issue is further discussed below in a section titled “Limitations.”

The primary purpose of this experiment was to assess the utility of the CORT paradigm as a preliminary screen for changes in membrane protein clustering that might be good candidates for additional assessment in tissue samples from human patients. The evaluation of membrane protein clusters in lymphocytes have specific technical conditions that should be considered in terms of replicability of results. The time, concentration, and nature of the fixative, and the temperature and incubation time of lymphocytes with the primary antibody are crucial factors to be considered because they will affect the reorganization of the lipid rafts and also the quality and resolution of the staining. In this experiment we used the same protocol already presented in previous publications from our group (Rivera-Baltanas et al., 2010, 2012, 2014, 2015; Romay-Tallon et al., 2017). We analyzed the clustering of several neurotransmitter markers (transporters and receptors) as well as pannexin 1 and PrPc, on the cell membrane of peripheral blood lymphocytes. Immunolabeling of these proteins is observed on the membrane as microscopic dots (clusters) that are amenable quantification using images analysis programs (Romay-Tallon et al., 2017). Expression of these proteins has been repeatedly identified in lymphocytes populations (SERT: Marazziti et al., 1998; Gordon and Barnes, 2003; Barkan et al., 2004; Yang et al., 2007; Chen et al., 2015; Herr et al., 2017. 5HT2A: Padin et al., 2006; Yang et al., 2006. DAT: Marazziti et al., 2010; Buttarelli et al., 2011. b2AR: Fan and Wang, 2009; Sanders, 2012. NR2B: Miglio et al., 2005; Biermann et al., 2007. PNX1: Woehrle et al., 2010. PrPc: Isaacs et al., 2006).

The role of neurotransmitter transporters and receptors in the immune system seems to be mainly regulatory, although this is still unclear (Robson et al., 2017). For instance, monoamines regulate T lymphocyte activation and secretion of chemokines by neutrophils (Leon-Ponte et al., 2007; Maes et al., 2012); similarly, glutamate is modulating T lymphocyte proliferation (Miglio et al., 2005). Pnx1 regulates T-cell activation at the immune synapse (Woehrle et al., 2010), whereas PrPc also modulates T-cell activation and its cross-linking leads to rearrangement of components of lipid rafts translating into an increased phosphorylation of signaling proteins (Isaacs et al., 2006). Many studies have pointed out alterations in components in the immune system in major depression, and this has recently become a hot research topic particularly in relation to multiple evidences implicating an important role of inflammation in depression (see Miller and Raison, 2016), and the roles of serotonin in inflammation and immunity (recently reviewed by Wu et al., 2018). This, together with the demonstrated antidepressant effects of some anti-inflammatory drugs such as etanercept (Kappelmann et al., 2016; Brymer et al., 2018) brings about a novel interest in focussing on different components of the immune system as putative targets of novel antidepressant drugs.

Our analysis indicates that CORT brings about alterations in the pattern of membrane protein clustering, resulting in an increase in cluster size for all the proteins analyzed with the exception of β2AR, which shows a decrease in cluster size. In addition, cluster numbers tend to be unaltered by CORT treatment but they are increased in the case of Pnx1 and PrPc. As all these proteins tend to accumulate within the membrane domains of lipid rafts (see Magnani et al., 2004; Taylor and Hooper, 2006; Besshoh et al., 2007; Fallahi-Sichani and Linderman, 2009; Swanwick et al., 2009; Botto et al., 2014), the results could be interpreted as indicating that CORT treatment could alter protein clustering within lipid rafts, which may have important physiological/pathological consequences. Interestingly, most CORT induced alterations in membrane protein clustering tend to show a positive correlation with immobility in the FST, i.e., with a depressive phenotype. A series of elegant reports by Rasenick’s lab point out that the action of antidepressant drugs in modulating Gα protein redistribution in rafts and non-rafts domains, and suggest that this action may be of importance for the onset of antidepressant activity, in fact they propose that it is this modulation of Gα protein distribution in raft domains by antidepressant drugs what brings about alterations in G-protein functionality that underlie these drugs antidepressant effects more than these effects being mediated by the direct binding to specific neurotransmitter transporters (Allen et al., 2005, 2007, 2009; Donati and Rasenick, 2005; Sugama et al., 2007; Dave et al., 2009; Zhang and Rasenick, 2010; Czysz and Rasenick, 2013; Czysz et al., 2015; Erb et al., 2016). Considering this, it would thereby be of interest to evaluate whether antidepressant treatment is also able to reverse the alterations in membrane protein clustering in lipid rafts induced by CORT and if this correlates with a normalization of the depressive phenotype. In fact, preliminary data from our laboratory suggests that this is what is happening (Romay-Tallon, personal communication). It would also be of interest to analyze if similar alterations in the patterns of membrane protein clustering are also found in the CNS, while our preliminary studies also indicate that this is the case at least for SERT (Romay-Tallon, personal communication) additional experiments need to be performed to validate that this is so.

Previous studies from our laboratory have shown alterations in the size but not in the number of clusters that express SERT in patients with depression (Rivera-Baltanas et al., 2012, 2015). The analysis of SERT clustering in the repeated CORT model of depression reveals parallel alterations to those described in naïve depression patients (Rivera-Baltanas et al., 2012). More importantly, we have shown that alterations in SERT clusters size correlate with depressive-like behavior upon CORT treatment indicating that alterations in SERT clustering are related to the depressive phenotype. In a similar way, we have also shown that the therapeutic outcome of antidepressant treatment in depression can also be predicted by alterations in SERT clustering in lymphocytes in naïve patients and proposed that a SERT clustering assay could be used as a biomarker of therapeutic efficacy in major depression (Rivera-Baltanas et al., 2012, 2015). This brings about the interest to analyze in depression patients the patterns of MPC for the other proteins analyzed in this study, and strongly suggests that the repeated CORT model of depression could be used to screen MPC of multiple proteins previous to their analysis in depression patients. In fact, approaches using both animal models and samples from depression patients are becoming common place for the study and characterization of specific biomarkers (see as example Carrillo-Roa et al., 2017).

These series of hypotheses-based studies (bench to bedside to bench) initiated with the demonstration of altered patterns of SERT clustering in animals with low levels of reelin (Rivera-Baltanas et al., 2010) and the demonstration that those animals are more susceptible to some behavioral alterations induced by CORT (Lussier et al., 2011), followed by the demonstration of altered patterns of SERT and 5HT2A clustering in naïve depression patients and its proposal as putative biomarkers of therapeutic efficacy for major depression (Rivera-Baltanas et al., 2012, 2014, 2015). These experiments have been now followed with the demonstration of similar alterations in the CORT model of depression and thereby with the possible use of this model to screen for additional proteins to be further assayed in depression patients as putative biomarkers of depression. The use of this approach is perfectly compatible with the use of other “omics” approaches that are been widely assayed (i.e., genomics, proteomics, metabolomics), and it may well be that an algorithm encompassing several methodologies will be more effective for the developing of novel biomarkers with a clear clinical appeal, as for example it is currently been applied in cancer biomarkers research (see as review, Goossens et al., 2015).

## Limitations

The present report primarily focusses on analyzing if changes in the pattern of membrane clustering of SERT and 5HT2A are also observed in the CORT model of depression, and thereafter in the analysis of the patters on clustering of several additional proteins with the purpose of using this test for their screening and further analysis in depression patients. While this has been so, one should be aware of some limitations of the present study, some of those pertaining to technological aspects on identification and quantification of protein clusters have been discussed above, but there are remain other limitations that should be considered that mostly pertain to the validity of the CORT model of depression and the use of specific behavioral tests.

The CORT model of depression has been widely used and validated in multiple behavioral tests (see as a review Sterner and Kalynchuk, 2010), but as it happens with most animal models it has some shortcomings and thereby data obtained by studying this model should always be related to the real thing, in this case what happens with alterations in membrane protein clustering in lymphocytes in depression patients. In the present report we indicate that changes in the pattern of clustering in SERT and 5HT2A parallel those observed in naïve depression patients and thereby we furthered the study by analyzing changes in the patterns of clustering of other membrane proteins. Although we are proposing to use this approach to screen for proteins to be thereafter studies in blood samples from depression patients, we cannot at the moment ascertain that the alterations found for these proteins in the CORT model would also be observed in depression patients. In addition, it would also be of interest to provide data from other animal models of depression that would further justify the use of animal models for screening of patterns of membrane protein clustering to be later translated to analyses in samples from depression patients.

A second shortcoming related to the use of the FST to establish depressive-like behavior. As mentioned at the beginning of the discussion, the FST is better used for screening of antidepressant-like activity than for demonstration of depression-like behavior (Nestler and Hyman, 2010; Molendijk and de Kloet, 2015; de Kloet and Molendijk, 2016). For the present experiments the FST was used only to demonstrate an effect of CORT similar to that we have shown multiple times (reviewed in Caruncho et al., 2016), but the CORT model also results in other emotional and cognitive alterations that are part of a depressive-like phenotype (see Kalynchuk et al., 2004; Gregus et al., 2005; Marks et al., 2009; Brymer et al., 2018).

Although out of the scope of the present report we understand that it would be of interest to ascertain the effects of antidepressants in membrane protein clustering and how these effects correlate with a reversal of different behavioral components in several models of depression, we are currently carrying out several studies to further on the knowledge on this topic.

## Conclusion

We have observed that repeated CORT alters the clustering of several proteins that tend to accumulate within lipid rafts, and that these alterations correlate with depression-like behavior. Changes in membrane protein clustering parallel those observed in naïve depression patients, indicating a possible use of the repeated CORT model to screen clustering parameters in multiple proteins as a preliminary step to their analysis as biomarkers of therapeutic efficacy in human blood samples from major depression patients.

## Author Contributions

RR-T, JO, LK, and HC designed the experiments. RR-T, EK, KB, JA, and TR-B, carried out the experiments. RR-T and TR evaluated the statistics. All authors reviewed and discussed the complete set of data. RR-T wrote the original manuscript. LK and HC finalized the manuscript.

## Conflict of Interest Statement

The authors declare that the research was conducted in the absence of any commercial or financial relationships that could be construed as a potential conflict of interest.
